# Twin study reveals non-heritable immune perturbations in multiple sclerosis

**DOI:** 10.1038/s41586-022-04419-4

**Published:** 2022-02-16

**Authors:** Florian Ingelfinger, Lisa Ann Gerdes, Vladyslav Kavaka, Sinduya Krishnarajah, Ekaterina Friebel, Edoardo Galli, Pascale Zwicky, Reinhard Furrer, Christian Peukert, Charles-Antoine Dutertre, Klara Magdalena Eglseer, Florent Ginhoux, Andrea Flierl-Hecht, Tania Kümpfel, Donatella De Feo, Bettina Schreiner, Sarah Mundt, Martin Kerschensteiner, Reinhard Hohlfeld, Eduardo Beltrán, Burkhard Becher

**Affiliations:** 1 Institute of Experimental Immunology, University of Zurich, Zurich, Switzerland; 2 Department of Neurology, University Hospital Zurich, Zurich, Switzerland; 3 Institute of Clinical Neuroimmunology, University Hospital, LMU Munich, Munich, Germany; 4 Biomedical Center (BMC), Faculty of Medicine, LMU Munich, Martinsried, Germany; 5 Munich Cluster of Systems Neurology (SyNergy), Munich, Germany; 6 Department of Mathematics, University of Zurich, Zurich, Switzerland; 7 Department of Computational Science, University of Zurich, Zurich, Switzerland; 8 Department of Strategy, Globalization and Society, University of Lausanne, Lausanne, Switzerland; 9 Gustave Roussy Cancer Campus, Villejuif, France; 10 Institut National de la Santé Et de la Recherche Médicale (INSERM) U1015, Equipe Labellisée—Ligue Nationale contre le Cancer, Villejuif, France; 11 Singapore Immunology Network, A*STAR, Singapore, Singapore; 12 Present Address: Neurologic Clinic and Policlinic, University Hospital Basel, Department of Biomedicine, University of Basel, Basel, Switzerland

**Keywords:** Autoimmunity, Multiple sclerosis, Systems analysis, Risk factors

## Abstract

Multiple sclerosis (MS) is a chronic inflammatory disorder of the central nervous system underpinned by partially understood genetic risk factors and environmental triggers and their undefined interactions^1,2^. Here we investigated the peripheral immune signatures of 61 monozygotic twin pairs discordant for MS to dissect the influence of genetic predisposition and environmental factors. Using complementary multimodal high-throughput and high-dimensional single-cell technologies in conjunction with data-driven computational tools, we identified an inflammatory shift in a monocyte cluster of twins with MS, coupled with the emergence of a population of IL-2 hyper-responsive transitional naive helper T cells as MS-related immune alterations. By integrating data on the immune profiles of healthy monozygotic and dizygotic twin pairs, we estimated the variance in CD25 expression by helper T cells displaying a naive phenotype to be largely driven by genetic and shared early environmental influences. Nonetheless, the expanding helper T cells of twins with MS, which were also elevated in non-twin patients with MS, emerged independent of the individual genetic makeup. These cells expressed central nervous system-homing receptors, exhibited a dysregulated CD25–IL-2 axis, and their proliferative capacity positively correlated with MS severity. Together, our matched-pair analysis of the extended twin approach allowed us to discern genetically and environmentally determined features of an MS-associated immune signature.

## Abstract (web summary)

In monozygotic twins discordant for multiple sclerosis, the influence of genetic predisposition and environmental factors is determined using matched-pair analyses.

## Main

MS is the most common neurological disorder affecting young adults with rising global incidence and prevalence, particularly in Western countries^1^. Despite numerous genome-wide association and epidemiological studies^2–4^, the aetiology of MS remains largely unknown. Yet, environmental cues associated with increased risk of developing MS have been established, and include factors acting prenatally^5,6^, during adolescence and adulthood (for example, viral infections, low levels of vitamin D, and tobacco smoke)^7^. Conversely, a registry-based study investigated the familial recurrence rate of MS and revealed that monozygotic twin pairs displayed the highest familial risk (17% age-adjusted risk for the unaffected twin) of developing MS, indicating a strong heritable effect^8^. Accordingly, over 200 risk loci with moderate-to-subtle effects have been described, including HLA-DRB1*15:01, IL2RA and *IL7R* genes^3,9^. Given that only 18–24% of MS heritability can be explained by known risk loci^2,10^, it is evident that the aetiology of MS involves a complex interplay between polygenic risk variants and environmental triggers. Hence, investigating how genetic predisposition and environmental triggers shape the interactions of individual immune cells is vital to understand the pathophysiology of autoimmune diseases including MS.

Previous attempts linking individual risk factors with functional perturbation on a single-cell level have given some insight into the inflammatory processes underlying MS^8,11–13^. Similarly, several studies have compared the immune profiles of patients with MS to healthy individuals or other control individuals with a disease^14–16^. Whereas these cross-sectional analyses revealed potential biomarkers, they cannot control for the effects of the genetic heterogeneity underlying MS susceptibility required for a patient to develop MS and its potential influence on immune cell perturbations.

Here we conducted an in-depth pairwise analysis of the systemic immune compartment of 61 monozygotic twin pairs discordant for MS, in which both siblings carry the same genetic and early-life environmental risk for the disease, yet only one is affected by MS. This approach thus eliminated the majority of bias attributed to variable genetic and early environmental influences in a heterogenous population^17,18^. We combined the high-throughput of mass cytometry, facilitating the analysis of 57 twin pairs, with cellular indexing of transcriptomes and epitopes by sequencing (CITE-seq) of eight selected twin pairs to obtain a comprehensive overview of epitopes, transcriptome and T cell receptor (TCR) clonotypes. Substantialt MS-associated alterations were largely restricted to the myeloid and helper T (T_H_) cell compartments. Our study thereby resolves how the immune systems of twins, who share the same genetic and early environmental risk factors, can diverge towards distinct clinical phenotypes.

## MS signature in T_H_ and myeloid cells

**Fig. 1:**
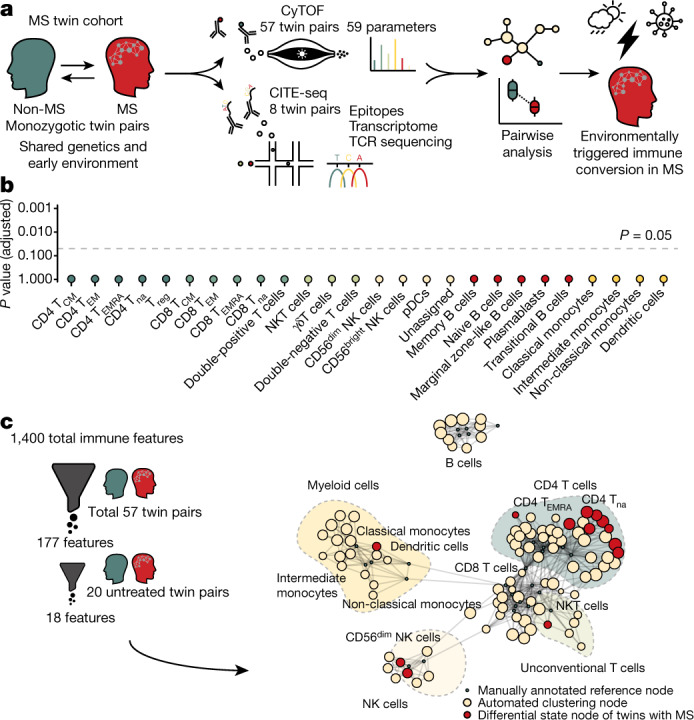
Twins discordant for MS exhibit differences in circulating T_H_ cell and myeloid compartments. **a**, PBMCs from monozygotic twin pairs discordant for MS were analysed by CyTOF (MS; *n* = 57) and CITE-seq (*n* = 8 + 2 healthy twins) to reveal an environmentally triggered immune conversion in MS. **b**, Lollipop plot showing *P* values for the two-sided paired non-parametric Wilcoxon signed-rank test with a false discovery correction according to the Benjamini–Hochberg approach of immune populations in the reference framework for untreated twins with MS (*n* = 20) and controls (*n* = 20). The dashed line indicates a 5% significance threshold. **c**, Automated analysis using diffcyt was performed by applying two filters screening for immune features that are different between all twin pairs (*n* = 57) and twin pairs in which the twin with MS remained untreated (*n* = 20) (left). Network visualization (right) of unsupervised FlowSOM clusters yielded by diffcyt analysis (beige; automated clustering nodes), the differential state nodes for twins with MS for which features appeared significantly different in both filters (red) and the nodes of the manually annotated reference framework defined in Extended Data Fig. 1c (green) are also shown. The dot size corresponds to the population frequency among total leukocytes. pDCs, plasmacytoid dendritic cells; T_eff_, effector T cell; T_na_, naive T cell; T_reg_, regulatory T cell.

We began by comparing the peripheral blood immune cell populations of 57 patients with MS and their unaffected monozygotic twin siblings (Extended Data Table 1, Supplementary Tables 1, 2). We applied cytometry by time-of-flight (CyTOF) to samples of peripheral blood mononuclear cells (PBMCs) to define the abundance of different cell types capturing 59 cellular parameters at the single-cell level (Fig. 1a, Supplementary Table 3). By combining data-driven and hypothesis-driven analyses, we created a cellular reference framework with well-defined canonical immune cell populations.

**Extended Data Fig. 1:**
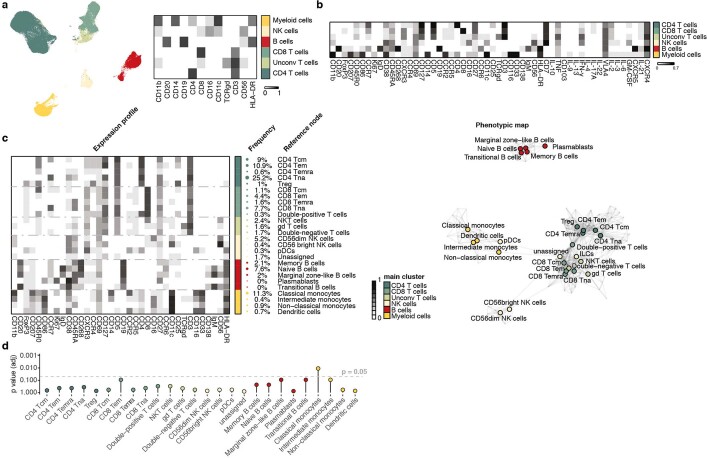
Changes in canonical immune composition in twin pairs discordant for MS can be attributed to disease-modifying treatment. **a**, UMAP of 1500 cells randomly sampled per patient from the MS twin cohort (left panel). Color code indicates FlowSOM clustering and manual annotation using the lineage markers presented in the heatmap (right panel). **b**, Heatmap showing the combined expression profiles of antigens analyzed by two partially-overlapping mass cytometry panels for the indicated immune populations. **c**, Expression profiles in the CyTOF dataset of the indicated markers across the immune cell populations associated with each reference node, alongside their relative mean population frequencies across all individuals in the MS twin cohort (n = 114). The FlowSOM-derived clusters were generated by iteratively clustering and manually merging the six main populations described in Extended Data Fig. 1a, b. Network representation of immune cell subsets in the manually-annotated reference framework based on phenotypic similarity (right panel). **d**, Lollipop plot showing p-values for the two-sided paired non-parametric Wilcoxon signed-rank test with a false-discovery correction according to the Benjamini-Hochberg approach of immune populations in the reference framework for all MS twins (n = 57) and controls (n = 57).

High-resolution clustering using FlowSOM and manual merging assigned all cells to a canonical immune subset, creating a framework of reference nodes that served as the basic substrate for the data-driven analysis (Extended Data Fig. 1a–c). Next, pairwise comparison of the frequencies of immune cell subsets in twins affected and unaffected by MS was performed, thereby controlling for the effects of genetic and shared early environmental (for example, prenatal and early childhood) influences. Although we uncovered significant differences in the frequency of classical monocytes in twins with MS (Extended Data Fig. 1d), we discovered that this was primarily driven by disease-modifying therapies (Fig. 1b). Thus, after eliminating genetic and early environmental sources of variance, monozygotic twins discordant for MS exhibit comparable frequencies of immune cell subsets in peripheral blood.

For a data-driven agnostic approach, which does not categorize cells into canonical subsets based on previous knowledge, the recently introduced diffcyt toolset based on the widely established Bayes moderated tests utilized in transcriptomic studies was applied. Markers were divided into lineage markers, which had served as the foundation for FlowSOM clustering, and cell-state markers including activation and trafficking molecules and cytokines (Supplementary Table 3). On the basis of this classification, diffcyt generated a list of 1,400 immune features, in which each feature represents the expression of a cell-state marker in a given immune cluster. Those features were then filtered by meeting two conditions: they distinguished twins with MS from their unaffected twin siblings across all twin pairs, and they were not elicited by disease-modifying therapy (Fig. 1c). Eighteen different immune features fulfilled both conditions in twins with MS and corresponding immune clusters were termed ‘differential state nodes’ (Extended Data Table 2). Twelve out of the 18 features localized within the T_H_ cell region, supporting previous observations^13,14^, one feature was close to the unconventional T cell reference nodes, three were close to the natural killer (NK) cell reference nodes and the remaining two were in the myeloid cell region (Fig. 1c, right). The most significant immune alterations were observed in the T_H_ cell and myeloid cell compartment (Extended Data Table 2). The conjunction of data-driven and previous knowledge-driven approaches has thus demonstrated that the clinical manifestation of MS, independent of the genetic predisposition, is predominantly determined in the T_H_ cell and myeloid compartments.

## CCR2–CSF2R are elevated in MS monocytes

**Fig. 2:**
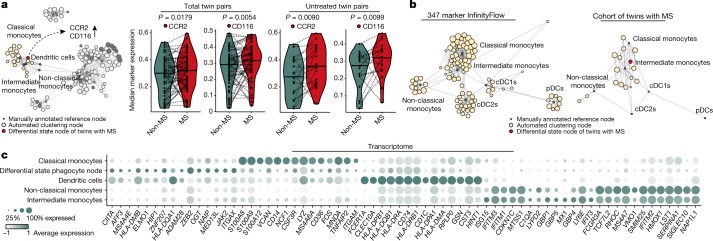
The myeloid landscape of twins with MS is shifted towards a monocyte population with increased expression of CCR2 and the GM-CSF receptor. **a**, Network visualization of unsupervised FlowSOM clusters in the myeloid compartment (beige); highlighted are the differential state nodes for twins with MS for which features appeared significantly different (red) and nodes of the manually annotated reference framework (green). The violin plots show the median expression levels of CCR2 and CD116 in monocytes within the differential state node from untreated twin pairs and all combined twins enrolled in the study; the bold horizontal line depicts the respective group mean and the dashed line indicates twinship. **b**, Mapping of diffcyt-generated myeloid cell clusters (right) on InfinityFlow data from PBMCs of a healthy donor outside the cohort of twins with MS (left). The dot size in **a**, **b** corresponds to the population frequency among total leukocytes. cDC1s, conventional type 1 dendritic cells. **c**, Dot plot of CITE-seq data (a total of 21,043 cells) showing the expression profile of the top 15 differentially expressed genes for each cluster. The dot size corresponds to the fraction of cells within each cluster expressing the indicated transcript, and the colour indicates average expression. If not indicated, the differences between the experimental groups were statistically not significant (*P* > 5%) using the moderated limma-trend method implemented in diffcyt, performing a false discovery correction according to the Benjamini–Hochberg approach.

**Extended Data Fig. 2:**
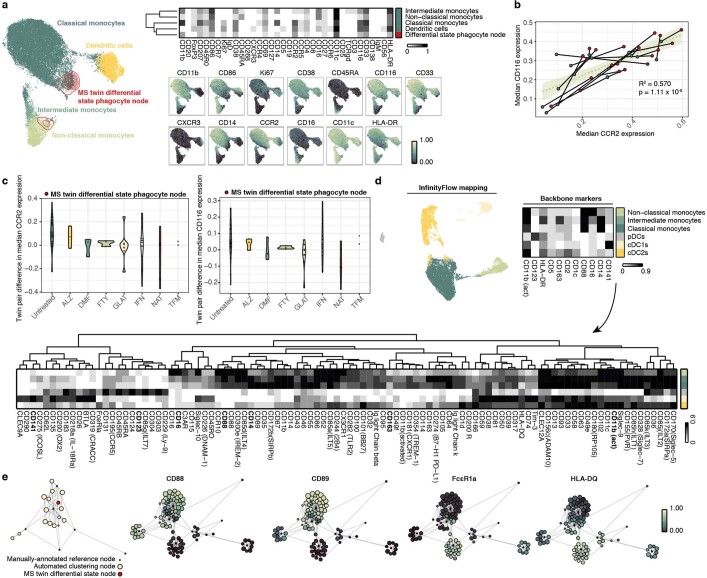
MS-affected twins show functional perturbations in the monocyte compartment. **a**, UMAP of 1500 randomly sampled myeloid cells per patient with color code indicating nodes of the myeloid reference nodes. Red color indicates density overlay of the differential state phagocyte node that showed increased CCR2 and CD116 expression in MS-affected compared to unaffected twins (left panel). Heatmap (top right panel) displays expression profile of differential state phagocyte node and reference nodes in phenotypic proximity. UMAP with expression overlay of markers present in the myeloid reference nodes (bottom right panel). **b**, 2D lollipop plot showing the median expression of CCR2 and CD116 levels in the differential state phagocyte node from unaffected twins (green) and MS-affected twins (red) that did not receive disease modifying treatment. Twin pairs are indicated by a solid line. Dashed line indicates smoothed conditional mean of the linear regression model with 95% confidence interval in shaded area. **c**, Violin plots showing the inter-twin-pair difference for median CCR2 (left panel) and CD116 (right panel) expression level in cells within the differential state phagocyte node, separated by treatment of the MS-affected twin. ALZ = Alemtuzumab (n = 3); DMF = Dimethyl Fumarate (n = 2); FTY = Fingolimod (n = 4); GLAT = Glatiramer Acetate (n = 8); IFN = Type 1 Interferons (n = 14); NAT = Natalizumab (n = 4); TFM = Teriflunomide (n = 2). **d**, UMAP of the myeloid cell compartment within healthy PBMCs outside the MS twin cohort from InfinityFlow data used to map the differential state phagocyte node of the MS twin dataset (top left panel). Color code indicates FlowSOM clustering and manual merging of the extended myeloid reference nodes by using the backbone markers presented in the heatmap showing the expression values (top right panel). The heatmap in the bottom panel displays the 100 most differentially expressed markers between the manually-annotated reference nodes. InfinityFlow backbone markers are presented in bold. **e**, Mapping of diffcyt-generated myeloid cell clusters in the MS twin dataset (left panel) on InfinityFlow data of healthy PBMCs (right panels) showing the overlay expression of CD88, CD89, FcεR1a, HLA-DQ. Dot size corresponds to population frequency among total leukocytes. Violin plots contain a bold horizontal line depicting the respective group mean. If not indicated, differences between experimental groups were statistically not significant (p > 5%) using a two-sided unpaired nonparametric Mann-Whitney-Wilcoxon test with a false-discovery correction according to the Benjamini-Hochberg approach.

Mononuclear phagocytes constitute a dominant fraction of leukocytes infiltrating the central nervous system (CNS) found in lesions of patients with MS^19,20^ and their inhibition has been reported to prevent neuroinflammation in mice^21–23^. Both MS-related features of the myeloid compartment were found in the same differential state node and exhibited higher levels of the receptor for monocyte chemoattractant protein 2 (CCR2) and the granulocyte–macrophage colony-stimulating factor (GM-CSF) receptor-specific subunit (CD116) in twins with MS (Fig. 2a). This differential state phagocyte node in twins with MS phenotypically resembled classical monocytes (Fig. 2a, Extended Data Fig. 2a). However, CD14 expression was dim and CD16 was absent, obstructing their unambiguous assignment to a canonical immune population (Extended Data Fig. 2a). The strong positive correlation between CD116 and CCR2 expression in monocytes within the differential state phagocyte node suggests a common regulation of both molecules (Extended Data Fig. 2b). Elevated expression of CCR2 and CD116 was especially pronounced in untreated twin pairs, suggesting that this particular MS-related perturbation is susceptible to disease-modifying therapies (Fig. 2a, right, Extended Data Fig. 2c).

To fully understand the identity of the cells within the differential state myeloid node, we enriched the cohort of twins with MS with InfinityFlow data of PBMCs for 347 detected surface markers derived from a healthy control sample outside the cohort of twins with MS^24^. InfinityFlow combines traditional flow cytometry-based detection of hundreds of marker molecules, with machine learning analysis, facilitating the mapping of cellular subsets with single-cell resolution^25^. We applied FlowSOM clustering to identify the canonical myeloid cell subsets of the peripheral blood and retrieved their surface proteome (Extended Data Fig. 2d). We next projected the differential state phagocyte node from the twin pairs with MS onto this map and identified their surface profile to be most similar to the classical monocyte and intermediate monocyte nodes (Fig. 2b, Extended Data Fig. 2e).

**Extended Data Fig. 3:**
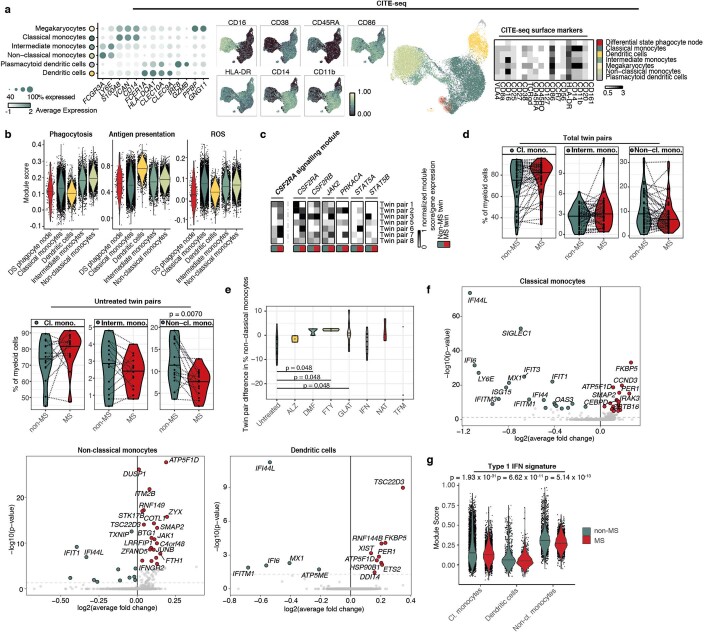
Transcriptional profiling of phagocytes of MS -affected twins and unaffected twin siblings. **a**, Dot plot showing the expression profile of selected signature markers in CITE-seq data of phagocytes derived from 7 twin pairs discordant for MS (21 043 cells; left panel). Dot size corresponds to the fraction of cells within each cluster expressing the indicated transcript and color indicates average expression. UMAP showing (middle panel) the expression overlay of indicated markers and cell type yielded by clustering. Heatmap showing the expression profiles of the indicated clusters determined by CITE-seq (right panel). **b**, Violin plots (of 20 678 cells) showing the module score for genes involved in phagocytosis, antigen presentation and reactive oxygen species (ROS) production for the indicated phagocyte clusters. **c**, Heatmap showing the module score for genes involved in *CSF2RA* signaling (left box) and normalized expression of individual selected genes involved in *CSF2RA* signaling (right box) for individual MS-affected and unaffected twins in the differential state phagocyte node (162 cells). **d**, Violin plots showing the frequency of manually-annotated reference nodes in proximity to the differential state phagocyte node determined by mass cytometry for all twin pairs (n = 57) and untreated twin pairs (n = 20). **e**, Violin plots showing the inter-twin-pair difference in non-classical monocyte frequency, separated by treatment of the MS-affected twin. **f**, Volcano plots showing the differentially expressed genes for the indicated clusters (a total of 18 024 cells) of MS-affected twins compared to unaffected twin siblings. The top 20 significant genes were annotated. Red color indicates increased and green color decreased gene expression in MS twins. **g**, Violin plots of CITE-seq data (a total of 18 024 cells) showing the module score of individual cells for type 1 IFN signature in clusters for which significant differences between MS-affected and unaffected twins were identified. Violin plots contain a bold horizontal line depicting the respective group mean. If not indicated, differences between experimental groups were statistically not significant (p > 5%) using a two-sided paired non-parametric Wilcoxon signed-rank test (**d**) or two-sided unpaired non-parametric Mann-Whitney-Wilcoxon test (**e**; both applying a false-discovery correction according to the Benjamini-Hochberg approach) or using a logistic regression model with the twinship as a latent variable and comparing each model to a null model with a likelihood ratio test and applying a Bonferroni correction (**f**, **g**).

To gain further insight into the functional properties of the differential state phagocyte node, we performed CITE-seq of the myeloid compartment of monozygotic twin pairs discordant for MS and obtained similar populations as present in the CyTOF data (Extended Data Fig. 3a, Extended Data Table 1). CITE-seq confirmed that the phagocyte node characterized by the CD14^dim^CD16^−^ phenotype showed transcriptional similarity to both classical monocytes and dendritic cells, as well as being characterized by the expression of *CIITA*, *ZEB2*, *JAK2* and *ITGAX* (Fig. 2c, Extended Data Fig. 3a, b). In addition, we observed a trend towards increased signalling activity of *CSF2RA* with increased expression of *CSF2RB*, *PRKACA* and *STAT5A* transcripts in twins with MS compared with their unaffected twin siblings (Extended Data Fig. 3c).

Exploring the monocyte reference nodes in proximity to the differential state node in the cohort of twins with MS, we further revealed that the frequency of non-classical monocytes among total myeloid cells was significantly reduced in twins with MS compared with their unaffected twin siblings. Again, this difference was more pronounced in untreated twin pairs (Extended Data Fig. 3d), and indeed treatment with dimethyl fumarate, fingolimod or glatiramer acetate significantly reduced the disparity between twins with MS and their unaffected twin siblings (Extended Data Fig. 3e). Finally, investigation of the transcriptional circuits, which differentiate twins with MS from unaffected twin siblings in the reference nodes of the myeloid compartment, revealed increased expression of *FKBP5*, *CCND3*, *PER1* and *IRAK3* in classical monocytes of twins with MS compared with their unaffected twin siblings (Extended Data Fig. 3f, Supplementary Table 4). Moreover, we observed an overall reduction in the type 1 interferon gene signature in classical monocytes, dendritic cells and non-classical monocytes of twins with MS compared with twins without MS, providing a potential link towards the clinical efficacy of recombinant interferon-β therapy in MS^26^ (Extended Data Fig. 3f, g, Supplementary Table 4).

In conclusion, twins with MS manifested a population shift in the myeloid compartment, away from non-classical monocytes and towards their inflammatory classical monocyte counterparts with a concomitant decrease in the type 1 interferon gene signature. A subpopulation of monocytes exhibited elevated expression of CCR2 and the GM-CSF receptor, indicative of a sensitization towards inflammatory stimuli.

## T_H_ cells display increased CD25 in MS

**Fig. 3:**
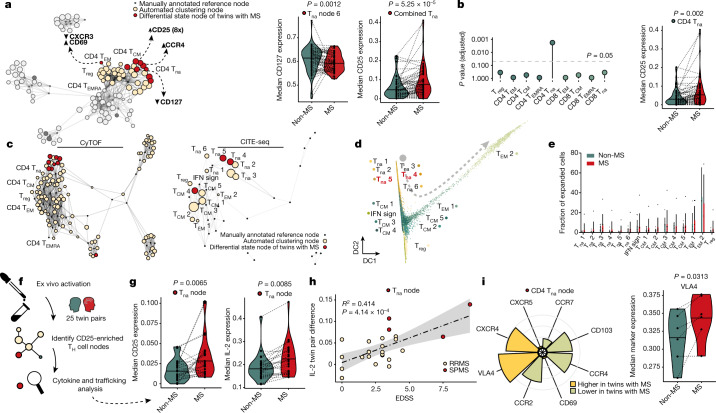
Transitional T_H_ cells of twins with MS display IL-2 hypersensitivity and elevated expression of brain-homing markers compared with unaffected twin siblings. **a**, Network visualizations of T_H_ cell clusters yielded by diffcyt analysis; highlighted are the differential state nodes for twins with MS for which features appeared significantly different (red, differential markers annotated in bold, 8x indicates number of differential nodes for the same feature) and nodes of the manually annotated reference framework (green). The violin plots show the median expression level for CD127 in cells within the differential state T_na_ 6 node and the median expression level for CD25 within the combined nodes for all twin pairs. **b**, Lollipop and violin plots showing the median expression levels of CD25 and the resulting *P* values across all T cell nodes in the reference framework of the naive T_H_ cell compartment for twin pairs discordant for MS (*n* = 57). **c**, Mapping of CITE-seq nodes (78,531 cells) from eight twin pairs discordant for MS onto the mass cytometry dataset. The red nodes indicate significantly increased surface expression of CD25 between twin pairs. **d**, Diffusion map showing the trajectory of T_H_ cells. The arrow indicates the direction of trajectory and nodes highlighted in red demonstrated increased CD25 expression in twins with MS. DC, diffusion coefficients. **e**, Bar graph showing the fraction of expanded T cell clones (*n* > 2 per clonotype) for twins discordant for MS (78,531 cells). **f**, PBMCs were activated in an antigen-independent manner, CD25-enriched T_H_ cell nodes were identified and analysed with regards to their cytokine and the trafficking profile in twin pairs discordant for MS (*n* = 25). **g**, Violin plot showing the median expression levels of CD25 and IL-2 in cells within the differential state nodes in twins with MS for all twin pairs. **h**, Correlation between the inter-pair difference in median expression of IL-2 and expanded disability status scale (EDSS) of twins with MS. RRMS is shown in beige (*n* = 21), and secondary progressive MS (SPMS) is shown in red (*n* = 4). The dashed line indicates the smoothed conditional mean of the linear regression model with a 95% confidence interval in the shaded area. **i**, Effect size for the expression of the indicated trafficking molecules (left) and a violin plot showing the median expression level of VLA4 (right) in cells within the differential state T_H_ cell node of twin pairs discordant for MS (*n* = 6). The dot size of the networks corresponds to the population frequency among total leukocytes. In the violin plots, a bold horizontal line depicts the respective group mean and the dashed line indicates twinship. If not indicated, the differences between experimental groups were statistically not significant (*P* > 5%) using the moderated limma-trend method implemented in diffcyt (**a**, left) or a two-sided paired non-parametric Wilcoxon signed-rank test (**a**, right, **b**, **g**, **i**), both applying a false discovery correction according to the Benjamini–Hochberg approach or a paired two-way analysis of variance (ANOVA) (**e**).

**Extended Data Fig. 4:**
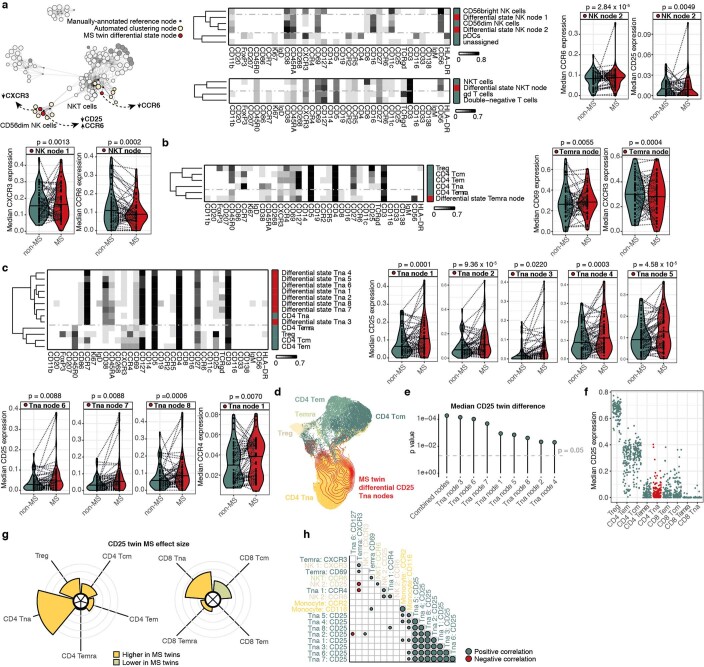
MS-affected twins are characterized by a transitional Th cell node displaying increased CD25 expression. **a**, Network visualization of unsupervised FlowSOM clusters in the unconventional T cell and NK cell compartment (left panel) yielded by diffcyt analysis (beige; automated clustering nodes); highlighted are MS twin differential state nodes for which features appeared significantly different in both filters (red) and nodes of the manually-annotated reference framework (green). Dot size corresponds to population frequency among total leukocytes. Heatmap showing the expression profile of MS twin differential state nodes for immune features present in the unconventional T cell and NK cell compartment and manually-annotated reference nodes in phenotypic proximity (middle panel). Violin plots showing the indicated median marker expression level in cells within the differential state NK and NKT cell nodes for all twin pairs enrolled in the study (n = 57; right panel). **b**, Heatmap showing the expression profile of MS twin differential state Temra node and manually-annotated reference nodes in phenotypic proximity (left panel). Violin plots showing the median CD69 and CXCR3 expression level in cells within the differential state Temra nodes for all twin pairs enrolled in the study (n = 57; right panel). **c**, Heatmap showing the expression profile of MS twin differential state nodes for immune features present in the naïve Th cell compartment and manually-annotated reference nodes in phenotypic proximity (left panel). Violin plots showing the median CD25 and CCR4 expression level in cells within the differential state naïve Th cell nodes for all twin pairs (right panel). **d**, UMAP of 1500 randomly sampled Th cells per patient with color code indicates FlowSOM clustering and manual merging of the Th cell reference nodes presented in Extended Data Fig. 1c. Red color indicates density overlay of the differential state naïve Th cell nodes that showed significantly increased CD25 expression between MS-affected twins and unaffected twin siblings. **e**, Lollipop plot showing p-values for the median CD25 expression levels across all differential state naïve Th cell nodes individually and combined for all MS-affected twins (n = 57) and unaffected twin siblings (n = 57; left panel). **f**, Dot plot showing the median CD25 expression level among cells within all T cell reference nodes for all patients enrolled in the study (n = 114). **g**, Effect size for the twin-pair difference in median CD25 expression across cells from the manually-annotated reference Th cell (left panel) or cytotoxic T cell nodes (right panel) for all twin pairs (n = 57). **h**, Correlogram showing the correlations between significant immune features for all individuals (n = 114). Dot size indicates the magnitude of correlation coefficient, green dots indicate a positive correlation and red dots a negative correlation. Violin plots contain a bold horizontal line depicting the respective group mean and dashed line indicates twinship. If not indicated, the differences between experimental groups were statistically not significant (p > 5%) using a two-sided paired non-parametric Wilcoxon signed-rank test (**e**, **g**) or using the moderated *limma-trend* method implemented in *diffcyt* applying a false discovery correction according to the Benjamini-Hochberg approach (**a**–**c**).

The unbiased feature extraction revealed the most prominent perturbations between twins with MS and unaffected twins within the lymphocyte compartment (Fig. 1c, Extended Data Table 2). Within the innate lymphocyte compartment, we observed increased expression of CCR6 and reduced amounts of CXCR3 and CD25 in NK cells and lower levels of CCR6 expression in an NKT cell node of twins with MS (Extended Data Fig. 4a). Significantly lower expression of CXCR3 and higher expression of CD69 were found in a node of CD4^+^ effector memory T cells re-expressing CD45RA (T_EMRA_) in twins with MS (Fig. 3a, Extended Data Fig. 4b).

However, the most dominant perturbations across our twin cohort were present in T_H_ cell nodes: we observed significantly higher expression of CD25, the IL-2 receptor high-affinity chain, in eight clusters phenotypically characterized as naive T_H_ cells that was accompanied by increased expression of CCR4 and decreased expression of CD127 in one node, respectively (Fig. 3a, Extended Data Fig. 4c). Although the overall expression of CD25 across naive T_H_ cells was relatively low, reducing the signal-to-noise ratio of the twin setup combined with barcoding and simultaneous acquisition revealed a robust increase in the expression of CD25 in twins with MS. This perturbation was the statistically most substantial change in the data-driven immune profiling of the cohort of twins with MS. CD25 represents a major genetic MS risk allele^27^. It is therefore of great interest that the unbiased analysis of twin siblings discordant for MS, with a shared genetic makeup, identified CD25 expression as one of the main features of the differential state node in T_H_ cells in siblings with MS.

When we mapped the eight differential state naive T_H_ cell nodes characterized by elevated expression of CD25 onto the reference T_H_ cell nodes for twins with MS, we found that they were phenotypically similar (Extended Data Fig. 4c, d). Binning of the eight clusters into a single naive T_H_ cell node also further increased the pairwise significance of the inter-twin difference in CD25 expression (Extended Data Fig. 4e). Investigation of our manually annotated canonical T cell reference nodes revealed that the expression of CD25 was low in naive T_H_ cells. However, the expression of CD25 in naive T_H_ cells was still higher than cytotoxic T cells (Extended Data Fig. 4f). Although significant differences in the expression of CD25 between pairs of twins with MS and their unaffected twins were restricted to T_H_ cells displaying a naive phenotype, the most marked differences were limited to a very discrete set of T_H_ cell nodes (Fig. 3b, Extended Data Fig. 4g).

Correlation analysis of significantly dysregulated immune traits in the cohort of twins with MS revealed that CD25 features in naive T_H_ cells not only strongly correlated with each other but also with the expression of CD116 and CCR2 in monocytes, suggesting interconnectivity between these features during immunopathology (Extended Data Fig. 4h). Together, elevated expression of CD25 within a subgroup of phenotypically naive T_H_ cells represented the most pervasive immune alteration in circulating immune cells of twins with MS.

## CD25 affects transitional T_H_ cells in MS

To further expose the cellular identity of the CD25 differential state T_H_ cell nodes in twins with MS, we performed CITE-seq, thereby retrieving the single-cell transcriptome in combination with single-cell TCR and surface epitope information.

**Extended Data Fig. 5:**
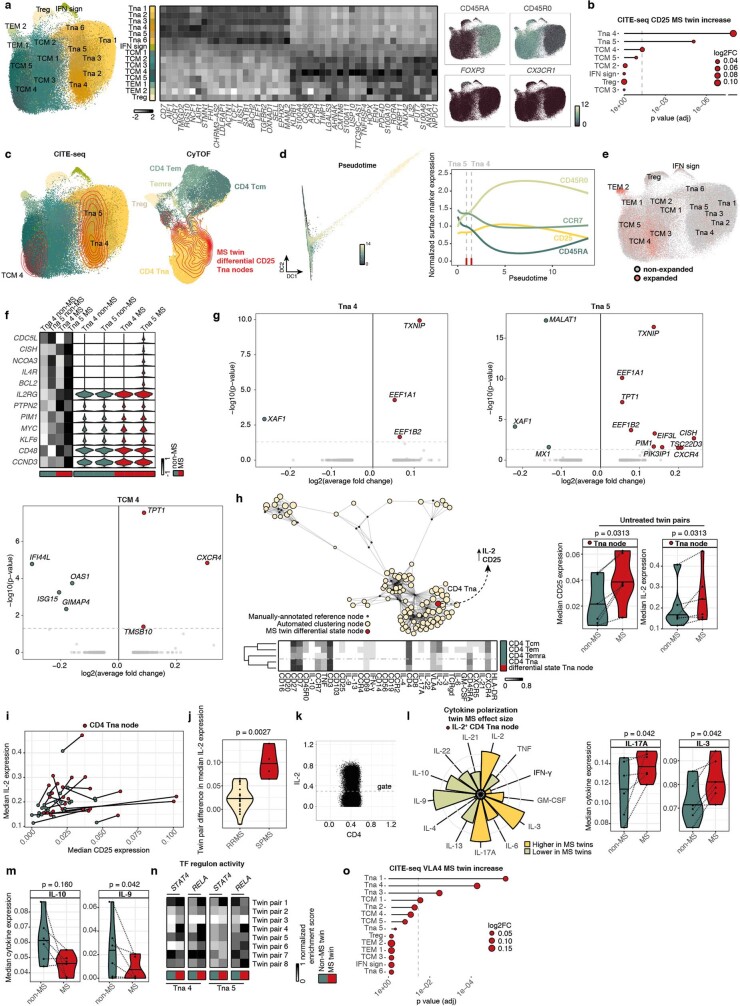
Transitional Th cells from MS-affected twins respond with increased IL-2 expression upon ex vivo reactivation. **a**, UMAP showing CITE-seq data of Th cells (78 531 cells) derived from 8 twin pairs discordant for MS (left panel). Color coding indicates cell type yielded by clustering using the transcriptome. Heatmap showing the expression profile of the top differentially expressed genes across the different clusters (middle panel). UMAP showing the expression overlay for surface CD45RA and CD45R0 and *FOXP3* and *CX3CR1* transcripts. **b**, Lollipop plot showing p-values for increase in CD25 surface epitope expression between MS-affected twins and unaffected twins for the indicated clusters determined by CITE-seq (45 146 cells). **c**, UMAP of CITE-seq and CyTOF data with an overlay showing the transitional Th cell nodes that demonstrated a differential CD25 expression in MS-affected versus unaffected twins. **d**, Diffusion map Th cells (78 531 cells) with pseudotime overlay (left panel). Line plot (right panel) showing the indicated surface markers expression across the computed pseudotime. Horizontal lines indicate mean pseudotime for Tna 4 and Tna 5 respectively. **e**, UMAP of CITE-seq data (78 531 cells) showing expanded cells (orange; > 2 TCR sequences in same patient) and non-expanded cells (grey). **f**, Heatmap and violin plots of selected genes within the IL2RA signaling pathway in Tna 4 and Tna 5 (11 343 cells) for MS-affected and unaffected twins. **g**, Volcano plots showing the differentially expressed genes of the indicated clusters (16 439 total cells) of MS-affected twins compared to unaffected twin siblings. The top 20 significant genes were annotated. Red color indicates increased and green color decreased gene expression in MS-affected twins. **h**, Network visualization (top left panel) of unsupervised FlowSOM clusters (beige); highlighted are MS twin differential nodes for which CD25 and IL-2 expression appeared significantly (red) and nodes of the manually-annotated reference framework (green). Dot size corresponds to population frequency among total leukocytes. Heatmap (bottom panel) displays marker expression profile of differential state naïve Th cell node and manually-annotated reference nodes in phenotypic proximity. Violin plots (right panel) showing the median CD25 and IL-2 expression levels in cells within the differential state naïve Th cell nodes in untreated twin pairs (n = 6). **i**, 2D lollipop plot showing the median expression of CD25 and IL-2 for unaffected twin siblings (green) and MS-affected twin siblings (red; n = 25). Twin pairs are indicated by solid line. **j**, Violin plots showing the inter-twin-pair difference in median IL-2 expression level in the differential state naïve Th cell node, separated according to disease stage of the MS-affected twin. RRMS = relapsing-remitting MS (n = 21); SPMS = secondary progressive MS (n = 4). **k**, Biaxial plot showing the IL-2 and CD4 expression levels for cells in the differential state naïve Th cell node. The dashed line indicates the gate to determine IL-2-positive cells. **l, m**, Effect size for the twin-pair difference of indicated cytokines in IL-2 expressing cells in the MS twin differential state node for untreated twin pairs (n = 6; **l**, left panel). Violin plots showing the median IL-17A and IL- 3 (**l**, right panel) or IL-10 and IL-9 (**m**) expression level in IL-2-expressing cells within the differential state transitional Th cell node for MS-unaffected twins and untreated MS-affected twins. **n**, Heatmap displaying the transcription factor regulon activity for *STAT4* and *RELA* in Tna 4 and Tna 5 (11 343 cells) determined by CITE-seq for individual twins. **o**, Lollipop plot showing p-values for increase in VLA4 surface epitope expression between MS-affected and unaffected twins for the indicated clusters (75 856 cells) determined by CITE-seq. Violin plots contain a bold horizontal line depicting the respective group mean and dashed line indicates twinship. If not indicated, the differences between experimental groups were statistically not significant (p > 5%) using an two-sided unpaired nonparametric Mann-Whitney-Wilcoxon test (**j**) or a two-sided paired non-parametric Wilcoxon signed-rank test (**h**, **l**, **m**) with false discovery correction according to the Benjamini-Hochberg approach or a logistic regression model using the twinship as a latent variable and comparing each model to a null model with a likelihood ratio test and applying a Bonferroni correction (**b**, **g**, **o**).

We identified six T_H_ cell clusters displaying a naive phenotype based on RNA (*CCR7*, *CD7*, *TCF7*, *LEF1* and *SELL*) and epitope expression (CD45RA^+^ and CD45R0^low^) (Extended Data Fig. 5a). CITE-seq confirmed elevated surface expression of CD25 in two clusters of naive T_H_ cells (T_na_ 4 and T_na_ 5), and a cluster of central memory T (T_CM_) cells (T_CM_ 4) in twins with MS compared with unaffected twins (Extended Data Fig. 5b). These clusters mapped in close proximity to the differential CD25 nodes of the mass cytometry dataset of twins with MS, thereby validating the high-throughput discovery CyTOF approach using an independent single-cell technology (Fig. 3c, Extended Data Fig. 5c).

Although the differential state T_H_ cell nodes in twins with MS displayed a naive phenotype, the increased surface expression of CD25 in twins with MS was indicative of cellular activation. Indeed, the transcriptomes of the T_na_ 4 and T_na_ 5 clusters placed these cells distant from bona fide naive T_H_ cells based on pseudotime analysis and both clusters started to downregulate naive markers (CD45RA and CCR7) while upregulating activation or memory-associated proteins (CD45R0 and CD25) (Fig. 3d, Extended Data Fig. 5d). Next, we investigated whether the increased expression of CD25 in the transitional T_H_ cell nodes of twins with MS is accompanied by an expansion of TCR clones. The fact that there was no indication for clonal expansion across T_H_ cells of twins with MS compared with unaffected twin siblings does not support the notion that the observed activation is driven by a shared autoantigen (Fig. 3e, Extended Data Fig. 5e).

To uncover the underlying transcriptional circuits that drive the difference in CD25 expression, gene expression of transitional T_H_ cell nodes was analysed. Apart from transcripts involved in IL-2 signalling such as *CISH*, increased expression of transcripts associated with protein synthesis and proliferation (*EEF1A1*, *EEF1B2*, *EIF3L*, *PIK3IP1* and *TPT1*) was found in twins with MS compared with unaffected twins (Extended Data Fig. 5f, g, Supplementary Table 5). The transcript showing the most significant induction in twins with MS compared with unaffected twins was *TXNIP*, which encodes thioredoxin-interacting protein (also known as vitamin D_3_ upregulated protein 1), a key mediator of the cellular antioxidant system that has been reported to regulate the responsiveness of T cells to IL-2 (ref. ^28^) (Extended Data Fig. 5g, Supplementary Table 5).

In summary, the unbiased single-cell transcriptome and epitope analysis of T_H_ cells across a selected group of twin pairs validated the specific increase of CD25 expression in a population of T_H_ cells in twins with MS initially captured by high-throughput mass cytometry. The transcriptome and pseudotime profiling further suggests that this population is within a transitional differentiation state with increased expression of genes related to protein biosynthesis and IL-2-induced proliferation.

## Cytokine dysregulation in MS progression

As CD25 has a crucial function regulating T cell proliferation and activation^29^, we next investigated the possible functional implications of increased expression of CD25 in the differential state transitional T_H_ nodes of twins with MS. Polyclonal stimulation of PBMCs from twin pairs discordant for MS using phorbol 12-myristate 13-acetate and ionomycin was followed by analysis of cytokines and trafficking-related markers (Fig. 3f). To anchor the CD25 differential state nodes in this additional dataset, a similar strategy as described above was applied. This revealed a T_H_ cell node displaying a naive phenotype characterized by increased expression of CD25 as well as its ligand IL-2 (Fig. 3g, Extended Data Fig. 5h, i). We observed a strong positive correlation between the size of inter-twin difference in IL-2 expression in the transitional T_H_ cell node and the severity of MS in the affected twin, assessed by the expanded disability status scale (Fig. 3h). Accordingly, the inter-twin difference in IL-2 expression was significantly higher in twin pairs in which the twin with MS had entered the more-advanced phase of disease progression known as secondary progressive MS, than twin pairs in which the affected twin was in a relapsing–remitting stage (RRMS) (Fig. 3h, Extended Data Fig. 5j).

To reveal early cytokine polarization across these transitional T_H_ cells, we gated on T_H_ cells that responded with IL-2 production to ex vivo reactivation (Extended Data Fig. 5k) and uncovered significantly lower production of IL-9 and higher production of IL-17A and IL-3 in twins with MS than in their unaffected twin sibling (Extended Data Fig. 5l, m). Both, IL-17A and IL-3 were reported to be secreted by encephalitogenic T cells in MS^30,31^, suggesting that these transitional, CD25-expressing, IL-2-producing peripheral T_H_ cells are the precursors to fully encephalitogenic T_H_ cells found in CNS lesions in patients with MS. To investigate putative transcriptional networks that drive this early polarization fate, DoRothEA, a computational tool to assess transcription factor activities based on reported transcription factor–target interactions, was used. Significantly higher *STAT4* activity in both transitional T_H_ cell nodes that demonstrated increased expression of CD25 (T_na_ 4 and T_na_ 5) coincided with elevated *RELA* activity in T_na_ 4 in twins with MS (Extended Data Fig. 5n, Supplementary Table 6). *RELA*, which encodes the NF-κB p65 subunit, has been shown to promote differentiation of IL-17-producing T cells^32^ and has been linked to MS in genome-wide association studies^33^. Similarly, mice deficient in STAT4 were resistant to experimental autoimmune encephalomyelitis, an animal model of neuroinflammation^34^.

## MS T_H_ cells display a trafficking signature

To follow the hypothesis that the proliferative T_H_ cell node in twins with MS could give rise to encephalitogenic T cells, we next measured the expression of trafficking molecules in the differential state T_H_ cell node. Compared with cells from the unaffected twin siblings, the cells from twins with MS expressed significantly higher amounts of VLA4, which was further validated using our CITE-seq dataset (Fig. 3i, Extended Data Fig. 5o). VLA4 is required for leukocyte migration into the CNS^35^ and is directly targeted by natalizumab, an approved therapy for MS^36^. Finally, *CXCR4* transcripts were enriched in the two T_H_ cell nodes that demonstrated increased expression of CD25 (T_na_ 5 and T_CM_ 4) in twins with MS compared with unaffected twins, further supporting the CNS trafficking potential of the identified transitional T_H_ cells (Extended Data Fig. 5g).

Together, in the peripheral blood of twins with MS, a population of transitional T_H_ cells is expanding due to increased expression of CD25. The level of expansion, determined by the production of IL-2 after ex vivo activation, correlates with disease severity. Expanding cells in twins with MS displayed early signs of encephalitogenic polarization, possibly driven by STAT4 and NF-κB activation, and demonstrated increased potential to traffic to the CNS.

## CD25 in naive T_H_ cells is a heritable trait

**Fig. 4:**
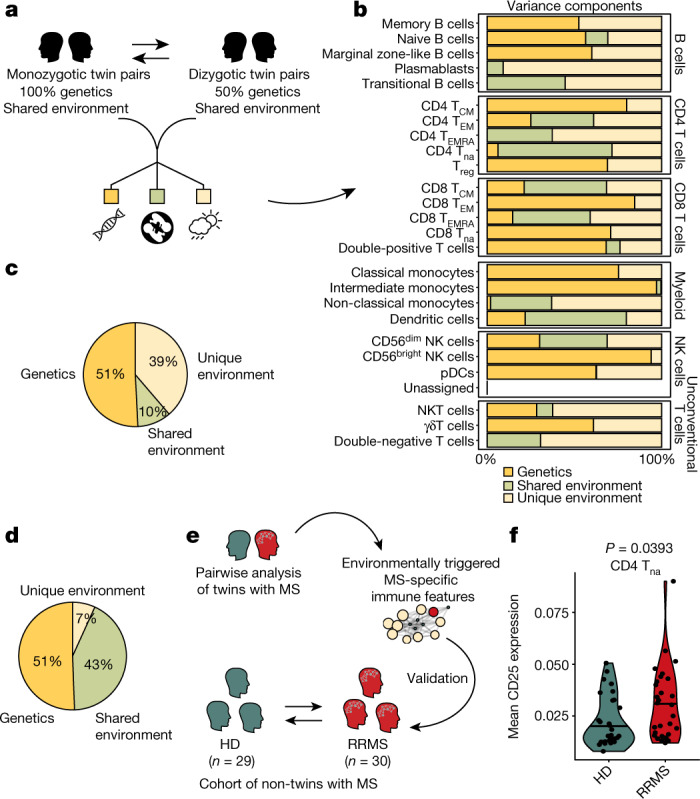
The expression of CD25 in naive T_H_ cells is regulated by genetic and early environmental factors and is increased in patients with MS in a cross-sectional validation cohort. **a**, Flow cytometry data of PBMCs from healthy monozygotic (*n* = 21) and dizygotic (*n* = 22) twin pairs age-matched to the cohort for twins with MS were used in a structural equation model to estimate the contribution of genetics and shared and non-shared environmental drivers on immune composition. **b**–**d**, Bar graphs (**b**) and pie charts (**c**, **d**) displaying the variance components for the populations in the manually annotated reference framework (**b**), the mean variance components across all detected immune subsets (**c**) and the variance components for the expression of CD25 in T_H_ cells displaying a naive phenotype (**d**). **e**, Data-driven analysis of twin pairs discordant for MS revealed MS-specific immune features that were validated within a cross-sectional validation cohort consisting of healthy donors (HD; *n* = 29) and patients with RRMS (*n* = 30). **f**, Violin plot showing the mean expression of CD25 in T_H_ cells displaying a naive phenotype of healthy donors and patients with RRMS. In the violin plots, the bold horizontal line depicts the respective group mean. If not indicated, differences between experimental groups were statistically not significant (*P* > 5%) using a two-sided unpaired non-parametric Mann–Whitney–Wilcoxon test with a false discovery correction according to the Benjamini–Hochberg approach.

**Extended Data Fig. 6:**
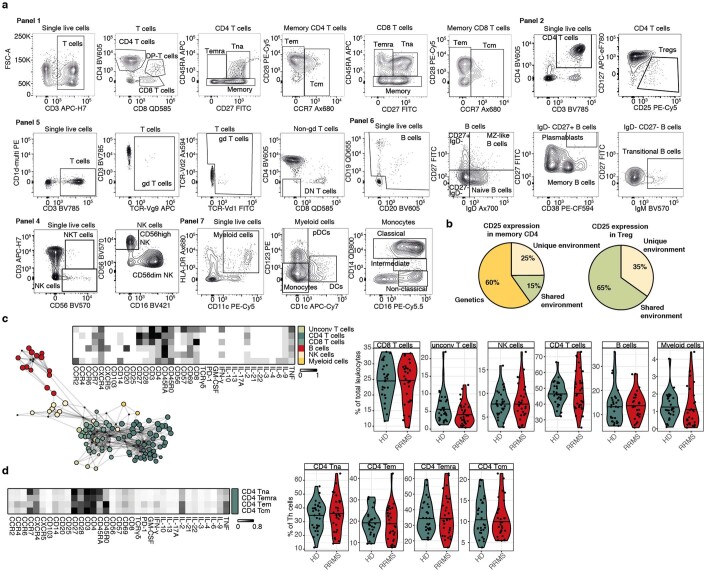
Monozygotic and dizygotic non-MS twin pairs facilitate dissection of genetic and environmental influences on immune composition. **a**, Biaxial plots displaying the gating strategy for cells from monozygotic and dizygotic twin pairs of the non-MS twin cohort in order to obtain comparable cell populations as present in the reference framework of the MS twin cohort. **b**, Pie chart displaying the variance components for CD25 expression in memory Th cells (left panel) and regulatory T cells (Treg; right panel) using the structural equation model and the median CD25 expression in the non-MS twin cohort. **c**, Heatmap (top left panel) displays expression profile of canonical subsets detected in the validation cohort. Network visualization of unsupervised FlowSOM nodes of the validation cohort and mapping onto the manually-annotated reference framework (grey dots; bottom left panel). Annotation provided in the publicly available mass cytometry dataset was used to match the main clusters in the reference nodes and indicated by the respective color. Dot size corresponds to population frequency among total leukocytes. Violin plots (right panel) showing the frequency of major canonical immune subsets in the validation cohort for HD (n = 29) and RRMS patients (n = 30). **d**, Violin plots showing the frequency of annotated Th cell nodes in the validation cohort for HD and RRMS patients (right panel). Heatmap (left panel) displaying the expression profile for the indicated Th cell populations. Violin plots contain a bold horizontal line depicting the respective group mean. If not indicated, differences between experimental groups were statistically not significant (p > 5%) using a two-sided unpaired nonparametric Mann-Whitney-Wilcoxon test with a false discovery correction according to the Benjamini-Hochberg approach.

Using the cohort of twins with MS, we were able to control for the genetic contribution to MS and thereby map immune alterations purely elicited by environmental cues. To extend this approach and estimate the respective effect of heritable, early environmental and late environmental influences on each immune trait in the reference framework, we next integrated flow cytometry data on PBMCs from a cohort of healthy monozygotic and dizygotic twin pairs (termed healthy twin cohort)^17^ that was age-matched to the cohort of twins with MS (Fig. 4a, Extended Data Table 1). Manual gating was used to match the cell populations from the healthy twin cohort to those in the reference framework of the cohort of twins with MS (Extended Data Fig. 6a). Using OpenMx, a structural equation model commonly utilized in twin studies, the extent to which each immune trait was modulated by genetic, early-shared or later unique environmental sources of variance during adulthood was estimated. The predominant sources of variance in immune cell frequencies differed between individual leukocyte subsets; although variance in frequency of CD56^bright^ NK cells and classical monocytes had a strong genetic component, plasmablasts and CD4^+^ T_EMRA_ cells were primarily influenced by unique environmental factors (Fig. 4b). Similar to previous reports^17,37,38^, we observed that approximately 50% of the variance across all identified immune populations was attributable to genetic influences, 40% to unique environmental factors and 10% to a shared early environment (Fig. 4c). Next, this method was applied to identify the influences associated with modulation of CD25 expression in T_H_ cells overlapping with a naive phenotype. In naive T_H_ cells, 51% of the variance in CD25 expression was regulated by genetics, 43% by shared (and thus early childhood) environmental factors and only 7% by unique environmental drivers (Fig. 4d, Extended Data Fig. 6b). Accordingly, analysis of the cohort of twins with MS eliminated 93% of the variance caused by heritable and early-childhood factors, thereby isolating disease-associated immune alterations that are unaffected by confounding variables.

## CD25 signature in non-twin patients with MS

Although the expression of CD25 in phenotypically naive T_H_ cells is largely regulated through heritable influences, the elevated expression of CD25 in transitional, quasi-naive T_H_ cells in MS is a disease-specific effect driven by unique environmental influences. To further solidify this concept, we analysed the expression of CD25 in a cross-sectional validation cohort of 30 untreated patients with RRMS and 29 genetically unrelated healthy donors^14^ (cohort of non-twins with MS) (Fig. 4e, Extended Data Table 1). Here the frequencies of canonical immune cell subsets were again comparable between patients with MS and non-familiarly related healthy donors^14^ (Extended Data Fig. 6c, d). Despite multiple sources of variation and the absence of enrichment for a familial MS susceptibility in controls of the cross-sectional cohort of non-twins with MS, we could observe that unfractionated naive T_H_ cells of patients with RRMS expressed higher levels of CD25 than healthy donors (Fig. 4f).

In conclusion, we show that elevated expression of CD25 in T_H_ cells that display a naive phenotype is part of the heritable (cross-sectional) and non-heritable (twins) immune signature of MS.

## Discussion

Here we used deep immune profiling of monozygotic twin pairs discordant for MS using two highly complementary single-cell technologies to begin to explain how two people sharing the same genetic predisposition for this disease can demonstrate such divergent clinical phenotypes. Data-driven analysis revealed that mainly the T_H_ cell and, to a lesser extent, the myeloid cell compartment were phenotypically altered in the systemic immune compartment of twins with MS. This confirmed and refined the findings of previous MS immunophenotyping studies, which could not control for the effects of genetic predisposition^13,14^. The observation that some of the findings revealed in this study have previously been reported in cross-sectional studies of MS (for example, increase in CCR2 expression in monocytes^39^), whereas other features demonstrated opposite trends of what has previously been described^40^, highlights the importance of discerning genetic predisposition from environmentally induced alterations in MS.

Compared with their unaffected twin siblings, the twins with MS exhibited a shift in their circulating monocyte compartment away from tissue-patrolling non-classical monocytes, and towards inflammatory monocytes. Analogously, ref. ^41^ reported that non-classical monocytes enter and patrol the CNS during the steady state, but are reduced and superseded by classical monocytes in the peripheral blood and cerebrospinal fluid of patients with RRMS. The role of inflammatory monocytes has been investigated intensively in the context of experimental autoimmune encephalomyelitis. Genetic ablation models have demonstrated that CCR2-expressing monocytes are the main executers of CNS immunopathology in this model, in which they sense T-cell-derived GM-CSF and adopt a pathological CNS-specific transcriptional signature that leads to tissue damage and neurological deficits^21,23,42^. We found that expression of both CCR2 and the GM-CSF receptor (CD116) was consistently increased in a subset of circulating monocytes in twins with MS. It is thus conceivable that T cell-orchestrated cytokine production leads to the observed phenotypic changes in the monocyte compartment, which may appear as an effect of the ongoing inflammation and ultimately contribute to tissue destruction in the CNS.

The most consistent pattern emerging from the pairwise analysis of monozygotic twins discordant for MS was significantly higher expression of CD25 in a population of T_H_ cells in patients with MS and their hyper-proliferative state. This exclusively affected a cluster of T_H_ cells transitioning from a naive to a memory or effector phenotype. Altered expression of CD25 in transitional T_H_ cells, predominantly under the influence of shared genetic and early environmental factors, highlights the crucial role of genetic predisposition in initiating MS. Despite eliminating the majority of heritable variance using the twin setting, the increase in the expression of CD25 in twins with MS still appeared as the most consistent immune dysregulation in the cohort of twins with MS. It is thus conceivable that polygenic risk variants, including single-nucleotide polymorphisms in the *IL2RA* gene^27,43^, confer genetic susceptibility that facilitates disease initiation by environmental challenges (for example, Epstein–Barr virus infection). Analogously, gene–phenotype correlations using clinically healthy high-risk allele carriers have revealed that the MS risk single-nucleotide polymorphism rs2104286 (in the *IL2RA* gene) resulted in increased expression of CD25 exclusively in naive T_H_ cells^44^. The postulated hypersensitivity for IL-2 in T_H_ cells in patients with MS has been shown to induce secretion of GM-CSF in high-risk allele carriers^11^, which represents a hallmark of CNS inflammation in both experimental autoimmune encephalomyelitis and human MS^14,45^.

Functionally, the increased expression of CD25 and IL-2 responsiveness of transitional T_H_ cells from patients with MS may provide the substrate for pathogenic CD4^+^ effector memory T cells in the inflamed CNS, as previously proposed^11,46^. This hypothesis aligns with the cytokine profile and expression of CNS-homing molecules such as CXCR4 and VLA4 in these T_H_ cells. Alternatively, homeostatically expanding CD25^high^ naive T_H_ cells^47^ found in the peripheral blood of individuals with MS might contribute, as bystanders, to an antigen-specific immune response occurring in secondary lymphoid organs or the CNS, amplifying cytokine production up to the threshold required to start or maintain the neuroinflammatory cascade of MS. In support of this notion, IL-2-mediated STAT5 signalling is sufficient to induce the production of GM-CSF in naive human T_H_ cells^11,46^. Accordingly, in the identified transitional T_H_ cell node, the increased expression of CD25 was accompanied by increased IL-2 in twins with MS and correlated with disease severity. Together with the changes in the composition of the circulating mononuclear phagocyte population, this indicates that a cytokine dysregulation characterized by activation of the IL-2RA–IL-2–GM-CSF axis, triggered by a unique environmental challenge, may well be the immunological substrate for disease initiation and/or progression in MS.

## Methods

### Sample selection for the cohort of twins with MS

The cohort of twins with MS is part of the MS TWIN STUDY and represents a cohort of monozygotic twins with discordance for MS and is located at the Institute of Clinical Neuroimmunology at the LMU Klinikum Munich, Germany. Recruitment started in May 2012 and is still ongoing; samples used in the present study were collected up to May 2020.

Inclusion criteria for study participation were met if in one twin of a monozygotic twin pair an diagnosis of MS according to the revised McDonald criteria^48,49^ was established, whereas the twin sibling was clinically healthy. Exclusion criteria were infection as well as treatment with antibiotics or high-dose intravenous glucocorticosteroids within 3 months before sampling. Monozygotic twin pairs clinically discordant for MS (*n* = 61) visited the outpatient department at the Institute of Clinical Neuroimmunology at the LMU Klinikum Munich for a detailed interview, neurological examination, blood sampling and MRI investigations (in a proportion of twins only). To confirm a diagnosis of MS, medical records including MRI scans were obtained and reviewed (Extended Data Table 1, Supplementary Tables 1, 2).

As the current disease-modifying treatment at the time of blood sampling is known to have a strong effect on the peripheral immune signature^38^, we selected a subgroup, in which the twin with MS had not received treatment at the time of blood sampling (*n* = 20) for further analyses (Extended Data Table 1, Supplementary Tables 1, 2). The expanded disability status scale (EDSS) was used as a measure of disease severity in twins with MS^50^.

The MS TWIN STUDY was approved by the local ethics committees of the Ludwig-Maximilians-University of Munich (ethics approval project number 267-13). All participants gave written informed consent, according to the principles of the Declaration of Helsinki.

### Blood sampling and PBMC preparation for the cohort of twins with MS

Blood samples of study participants of the MS TWIN STUDY were collected in EDTA-containing tubes. To exclude sample collection bias, blood samples were drawn from each twin pair before meals and at the same time on the same day. PBMCs were isolated as described before by density gradient centrifugation with Lymphoprep (STEMCELL technologies) and cryopreserved in liquid nitrogen using serum-free cryopreservation medium (CTL-Cryo ABC Media Kit, Immunospot) in concentrations of 1 × 10^7^ cells per ml.

### Ex vivo activation of PBMCs in the cohort of twins with MS

To measure cytokine expression by PBMC from the cohort of twins with MS, cells from 25 twin pairs (19 treated pairs and 6 untreated pairs) were activated in an antigen-independent manner as described previously^51^. In brief, leukocytes were taken from liquid nitrogen storage and thawed in a water bath at 37 °C. Cells were resuspended in cell culture medium (RPMI-1640, 10% FCS (Biochrom), 1× l-glutamine and 1× penicillin–streptomycin (both Life Technologies)) supplemented with 1:10,000 benzonase (Sigma-Aldrich), then centrifuged (350*g* for 7 min at 24 °C) and washed twice with cell culture medium. Samples subsequently underwent antibody labelling for mass cytometry, or, in the case of intracellular cytokine detection, were incubated overnight at 37 °C and 5% CO_2_, before stimulation with 50 ng ml^−1^ phorbol 12-myristate 13-acetate (Sigma-Aldrich) and 500 ng ml^−1^ ionomycin (Sigma-Aldrich) in the presence of 1× brefeldin A and 1× monensin (both BD Biosciences) for 4 h at 37 °C. Cells underwent surface marker antibody labelling, fixation, permeabilization and intracellular cytokine antibody labelling as described below.

### Antibodies

Antibodies used in mass cytometry experiments were either purchased already heavy-metal-conjugated (Fluidigm) or were conjugated in-house using the MaxPar X8 chelating polymer kit (Fluidigm) following the manufacturer’s instructions. Antibody clones, corresponding heavy metal tags and suppliers are summarized in Supplementary Table 3. Antibodies used in CITE-seq experiments were purchased preconjugated and are summarized in Supplementary Table 7.

### Live-cell barcoding for mass cytometry

To eliminate technical variability during sample processing and data acquisition, a restricted combinatorial 9-choose-3 live-cell barcoding strategy was applied, as described previously^14^. To achieve this, anti-CD45 monoclonal antibodies (mAbs; BioLegend) were conjugated using MaxPar X8 polymers (Fluidigm) and palladium (^104^Pd, ^105^Pd, ^106^Pd, ^108^Pd and ^110^Pd), indium (^113^In and ^115^In; all from Trace Sciences International) and tantalum (^181^Ta; Sigma) isotopes. In addition, Y89-conjugated anti-CD45 mAbs (Fluidigm) were used. Twin pairs were randomized and PBMCs of twins with MS and unaffected twin siblings were barcoded in two batches before acquisition during two independent mass cytometry runs. After sample thawing and/or ex vivo activation cells were labelled with heavy-metal-tagged CD45 antibodies at 37 °C for 25 min in cell labelling medium (CLM; RPMI-1640, 4% FCS) on an orbital shaker (500 rpm). Barcoded samples were washed twice in CLM and combined into a single-reaction vessel for surface marker and/or cytokine detection.

### Surface and intracellular cytokine detection by mass cytometry

After barcoding, the sample convolute was labelled in 400 μl CLM containing the antibody mix for surface marker detection (Supplementary Table 3) on an orbital shaker (500 rpm) at 37 °C for 40 min. Cisplatin (2.5 μM in PBS; Fluidigm) was added for 2 min on ice to enable live/dead cell discrimination and the reaction was stopped by adding 2% FCS in PBS and incubating for 2 min on ice.

For the detection of transcription factors, the barcoded sample convolute was fixed and permeabilized for 40 min at 4 °C in 1X FOXP3 fixation/permeabilization buffer (BioLegend) and was washed in permeabilization buffer (PBS, 0.5% saponin, 2% bovine serum albumin (BSA) and 0.01% sodium azide (all Sigma-Aldrich)). Labelling was performed in 400 μl permeabilization buffer containing the antibody mix for 1 h at 4 °C.

In case of intracellular cytokine detection, the sample convolute was fixed in 1.6% paraformaldehyde (Electron Microscopy Sciences) for 1 h at 4 °C and washed with permeabilization buffer. The sample was labelled with antibodies recognizing intracellular cytokines in 400 μl permeabilization buffer for 1 h at 4 °C.

For both surface and nuclear antigen detection and intracellular cytokine detection, the labelled sample convolute was washed and incubated in 1X iridium intercalator solution (Fluidigm) at 4 °C overnight. The sample convolute was washed twice with PBS and twice with MaxPar water (Fluidigm) and following data were acquired.

### Mass cytometry data acquisition and preprocessing

Data were acquired on a CyTOF 2.1 mass cytometer (Fluidigm) with daily instrument quality control and tuning. Acquisitions from two independent CyTOF runs, each containing both of the twin siblings, were normalized using five-element beads (Fluidigm)^52^. To monitor potential batch effects, each independent run contained two normalization control samples that were present in both runs. Manual gating using FlowJo (TreeStar) was applied to identify live single cells in the sample convolute based on event length, centre, width, DNA (^191^Ir and ^193^Ir) and live/dead (^195^Pt) channels. Following this, the sample convolute was debarcoded by utilizing Boolean gates of cells exclusively bearing three barcodes to prevent barcode mis-identification and facilitate doublet exclusion. Mass cytometry data were transformed in the R environment using an inverse hyperbolic sine (arcsine) function with varying cofactors to account for labelling variability between individual markers. To remove residual batch effects between the two independent mass cytometry runs, individual markers of the sample convolute were aligned by modifying the arcsine cofactor to achieve the same mean in labelling intensity for both normalization controls. A marker-based percentile normalization was applied using the 99.9th percentile of the transformed dataset. Analogously, individual cytokine positivity was determined by calculating the 99th percentile of the residual cytokine intensity using an unstimulated control.

### Algorithm-guided analysis of the cohort of twins with MS

The entire analysis was carried out in the statistical programming environment R in RStudio and Visual Studio Code (Microsoft). UMAPs were computed using the umap package with default parameters^53^. For the generation of the reference framework, FlowSOM was applied^54^ in a similar manner as described previously^51,55^. In brief, 100 clusters of the combined dataset were generated and metaclustering was performed based on the elbow criterion determined by the ConsensusClusterPlus package. Resulting metaclusters were manually merged and annotated based on the median expression profile of individual metaclusters and localization on the UMAP. After initial clustering yielded the main populations such as CD4^+^ T cells, CD8^+^ T cells, B cells and myeloid cells, each main population was iteratively clustered into subpopulations that depicted the nodes of the reference framework.

The data-driven analysis was carried out using the diffcyt package^56^. For this, markers were separated into cell-state and cell-type markers (Supplementary Table 3) and the differential state diffcyt analysis was carried out using a design matrix considering the paired design. Significant immune features between all twins with MS and unaffected twin siblings were extracted using the moderated limma-trend method implemented in diffcyt, applying a false discovery correction according to the Benjamini–Hochberg approach^57^. To exclude treatment-induced immune alterations, a second filter was applied to identify significant immune features between only untreated twins with MS and unaffected twin siblings. Accordingly, immune features were extracted that fulfilled both conditions: (1) they appeared significantly different between all twins with MS and unaffected twin siblings, and (2) were not elicited by disease-modifying therapy of the twin with MS.

Network visualizations to map diffcyt-generated clusters onto the reference framework or to map different mass cytometry panels, InfinityFlow data or CITE-seq data were generated using modifications of the grappolo and vite packages of the Scaffold framework^58^. Resulting force-directed graphs were rearranged using the Fruchterman–Reingold and Kamada–Kawai algorithms implemented in the igraph package and modified and visualized using ggraph. Heatmaps were drawn using the pheatmap package. Correlograms were generated using the Hmisc and corrplot packages. All remaining plots were drawn using ggplot2.

### Mapping of myeloid cell populations using InfinityFlow

To fine-map the differential state phagocyte node, an extended myeloid reference framework was generated using the InfinityFlow data of healthy PBMCs containing 14 backbone markers and 332 predicted markers as published by ref. ^24^. To generate the extended myeloid reference nodes, FlowSOM clustering was applied as described above using the backbone markers for clustering. The InfinityFlow dataset contained an overlap of 32 markers with the mass cytometry dataset of twins with MS that facilitated the mapping of the diffcyt-differential state node on the extended reference nodes using the grappolo and vite packages as described above.

### Sorting of twin samples and single-cell sequencing

Frozen PBMC samples from eight twin pairs with MS (16 samples; four pairs analysed by both CyTOF and CITE-seq and four additional pairs exclusively by CITE-seq) plus two additional healthy samples (total of 18 samples) were thawed quickly and washed twice with 1% BSA in PBS at 4 °C, followed by centrifugation at 300*g* for 10 min. The PBMC pellet was stained with Human TruStain FcX Fc Blocking Reagent (BioLegend) for 10 min and further stained with Fixable Viability Dye APC-eFluor 780, anti-CD3-AF700 (clone OKT3, Invitrogen), anti-CD4-pacific blue (clone S3.5, Invitrogen), anti-CD11c-PE (clone BU15, BioLegend), and TotalSeq-C antibodies (Supplementary Table 7) for 30 min on ice. CD4^+^ T cells were sort purified as a singlet, live, CD3^+^ and CD4^+^, on a FACSAria Fusion (BD). Simultaneously, a second population was sorted as a singlet, live, CD3^−^ and CD11c^+^.

Sorted cells were washed in 0.04% BSA in PBS. Approximately 25,000 cells per population per sample were loaded on a 10x chip and run onto the 10x Chromium controller using Chromium NextGEM Single Cell V(D)J Reagent kits v1.1 with Feature Barcoding technology for Cell-Surface Protein (10x Genomics) according to the manufacturer’s protocol. Gene expression, TCR enrichment and cell-surface protein expression were multiplexed using individual Chromium i7 Sample Indices. Gene expression and TCR enrichment libraries were sequenced on NovaSeq S4 Flowcells using 150-bp paired-end reads and 8 bp for the i7 index, aiming for 50,000 reads per cell for gene expression and 5,000 reads per cell for TCR enrichment. Cell-surface protein expression libraries were sequenced on NovaSeq S1 Flowcells using 50-bp paired-end reads and 8 bp for the i7 index, aiming for 10,000 reads per cell.

### Single-cell sequencing data processing

Cell Ranger software (10x Genomics, v.6.1) was used to demultiplex samples, process raw data, align reads to the GRCh38 human reference genome and summarize unique molecular identifier (UMI) counts. Filtered gene-barcode and cell-surface protein expression-barcode matrices that contained only barcodes with UMI counts that passed the threshold for cell detection were used for further analysis. Then, we processed the filtered UMI count matrices using the R package Seurat (version 4.0.3)^59^. Cells that expressed fewer than 500 genes and/or >15% mitochondrial reads, and genes expressed in fewer than three cells were removed from the count matrix. After quality control, only raw gene counts in high-quality singlets were submitted to: log-normalization; identification of high-variable genes by using the vst method; scaling; and regression against the number of UMIs and mitochondrial RNA content per cell. We applied an unbiased calculation of the *k*-nearest neighbours, generated the neighbourhood graph and embedding using UMAP. Differentially expressed genes between each cluster and all other cells were calculated using the FindAllMarkers function. Annotation of Seurat clusters was manually curated using a combination of upregulated genes for each cluster and visual inspection of key markers using UMAP visualization.

After initial cluster annotation, we subsetted all clusters containing myeloid cells and reanalysed this subset. After subsetting, integration using reciprocal principal component analysis was performed to remove batch effects and the integrated assay was used for principal component analysis and unsupervised clustering. Seurat subclusters were annotated using a combination of canonical protein and mRNA markers. Similar to myeloid cells, only CD4^+^ T cells in which a TCR clonotype was detected, were subsetted and reanalysed. Single-cell TCRs were computed from the TCR enrichment sequencing data using Cell Ranger vdj pipeline (10x Genomics, v.6.1). CD4^+^ T cells containing more than two β-chains were removed.

Only samples with data for both the sibling with MS and the unaffected sibling were used for downstream analysis: seven and eight twin pairs for myeloid and T_H_ cell populations, respectively.

### Downstream analysis of CITE-seq data

Differentially expressed genes between MS-affected and unaffected twin siblings were computed using a logistic regression model using the twinship as a latent variable. Mapping of CITE-seq data onto the mass cytometry data was accomplished by arcsine transformation of the raw counts obtained for each surface marker followed by percentile normalization similarly as performed for mass cytometry data. Resulting transformed and normalized data were combined with mass cytometry data to create a cellular network using the grappolo and vite packages as described above. Transcription factor regulon activity was inferred based on the gene expression levels of its targets using DoRothEA^60^. Trajectory and pseudotime were computed based on the corresponding UMAP using Monocle 3 (ref. ^61^). Subsequently, the Seurat object was converted into the .h5ad format for further trajectory analysis and calculation of diffusion maps using the SCANPY analysis framework implemented in Python^62^. Surface marker expression along pseudotime was smoothed using a generalized additive model from the mgcv package.

### Variance component analysis using the healthy twin cohort

To dissect genetic, early shared environmental and unique environmental sources of variance for frequencies of immune populations or median marker expressions within immune subsets, publicly available flow cytometry data from healthy monozygotic and dizygotic twin pairs were accessed^17^. Healthy monozygotic (*n* = 21) and dizygotic (*n* = 22) twin pairs were selected to be age-matched to the twin pairs in the cohort of twins with MS (Extended Data Table 1). A compensation matrix of the flow cytometry data was corrected using FlowJo (TreeStar) and populations matching the immune subsets of the reference framework in the cohort of twins with MS were retrieved using manual gating. Resulting immune subset frequencies or median marker expressions were imported into R. Variance components were estimated in a two-group Cholesky twin model using the umxACE function of the umx package applying default parameters—a framework commonly applied to twin studies^63,64^.

### Validation in the cross-sectional MS cohort

For the validation of the findings in the cohort of twins with MS, a publicly available cross-sectional cohort of non-twins with MS measured by mass cytometry was accessed^14^ (Extended Data Table 1). The analysis was carried out using exclusively untreated patients with RRMS. A main population and T_H_ cell subpopulation label for each cell was provided in the publicly available dataset.

### Statistical analysis

Immune cell frequencies and median marker expressions were compared using the unpaired non-parametric Mann–Whitney–Wilcoxon test or the paired non-parametric Wilcoxon signed-rank test implemented in the stats package with a false discovery correction according to the Benjamini–Hochberg approach^57^. For the validation cohort, the per-patient mean of CD25 expression was compared between patients with RRMS and health donors as a statistical parameter that is sensitive to outliers and could thus reflect variable CD25 expression by only a subset of naive T_H_ cells. Mass cytometry analysis was carried out in two independent runs and was not repeated due to limited precious sample material. CITE-seq analysis was carried out once. Gene expression, module scores and transcription factor activity between cells of twins with MS and unaffected twin siblings were compared using a logistic regression model with the twinship as a latent variable and comparing each model to a null model with a likelihood ratio test and applying a Bonferroni correction. Linear regression analysis was performed using the base lm function. Inter-twin pair effect sizes were computed using the Wilcoxon two-sample paired signed-rank test implemented in the rstatix package.

### Reporting summary

Further information on research design is available in the Nature Research Reporting Summary linked to this paper.

## Online content

Any methods, additional references, Nature Research reporting summaries, source data, extended data, supplementary information, acknowledgements, peer review information; details of author contributions and competing interests; and statements of data and code availability are available at 10.1038/s41586-022-04419-4.

### Supplementary information

Reporting Summary

Supplementary Table 1 **Clinical and demographic characteristics of all the patients in the MS twin cohort used for mass cytometry**. Disease state indicates whether the twin was affected by MS or unaffected. MS = Multiple Sclerosis; ALZ = Alemtuzumab; DMF = Dimethyl Fumarate; FTY = Fingolimod; GLAT = Glatiramer Acetate; IFN = Type 1 Interferons; NAT = Natalizumab; TFM = Teriflunomide; f = female; m = male; RRMS = relapsing-remitting MS; SPMS = secondary progressive MS; PPMS = primary progressive MS; EDSS = Expanded disability status scale; NA = not applicable; unk = unknown..

Supplementary Table 2 **Clinical and demographic characteristics of untreated patients in the MS twin cohort used for mass cytometry**. Disease state indicates whether the twin was affected by MS or unaffected. MS = Multiple Sclerosis; female; m = male; RRMS = relapsing-remitting MS; SPMS = secondary progressive MS; PPMS = primary progressive MS; EDSS = Expanded disability status scale; NA = not applicable; unk = unknown.

Supplementary Table 3 **Heavy metal labeled antibodies used in the mass cytometry experiments**. Metal tagged antibodies were either purchased preconjugated (Fluidigm) or conjugated using the MaxPar X8 polymer (for other suppliers). NA = non-applicable; ICS = intracellular cytokine staining.

Supplementary Table 4 **Differentially expressed genes in phagocyte clusters determined by CITE-seq**. Shown are cluster-gene combinations that were statistically significant (p adj < 0.05) comparing MS twins to their non-MS twin siblings using a logistic regression model with twinship as a latent variable, comparing each model to a null model with a likelihood ratio test and applying a Bonferroni correction. Pct indicates the fraction of cells within each cluster that expressed the respective gene. FC = fold change; pct = percent expressed.

Supplementary Table 5 **Differentially expressed genes in Th cell clusters determined by CITE-seq**. Shown are cluster-gene combinations that were statistically significant (p adj < 0.05) comparing MS twins to their non-MS twin siblings using a logistic regression model with twinship as a latent variable, comparing each model to a null model with a likelihood ratio test and applying a Bonferroni correction. Pct indicates the fraction of cells within each cluster that expressed the respective gene. FC = fold change; pct = percent expressed.

Supplementary Table 6 **Differential transcription factor activity in naive Th cell clusters determined by CITE-seq**. Shown are cluster-transcription factor combinations for which transcription factor activity was statistically significant (p adj < 0.05) comparing MS twins to their non-MS twin siblings using a logistic regression model with twinship as a latent variable, comparing each model to a null model with a likelihood ratio test and applying a Bonferroni correction. Transcription factor activity was estimated using DoRothEA, a tool that computes regulon activity based on reported transcription factor - target gene interaction. Pct indicates the fraction of cells within each cluster that were positive for the respective transcription factor activity. FC = fold change; pct = percent expressed.

Supplementary Table 7 **Oligonucleotide labeled antibodies used in the CITE-seq experiments**. Oligonucleotide tagged antibodies (TotalSeq-C) were purchased preconjugated from Biolegend. NA = non-applicable.

## Figures and Tables

**Extended Data Table 1:**
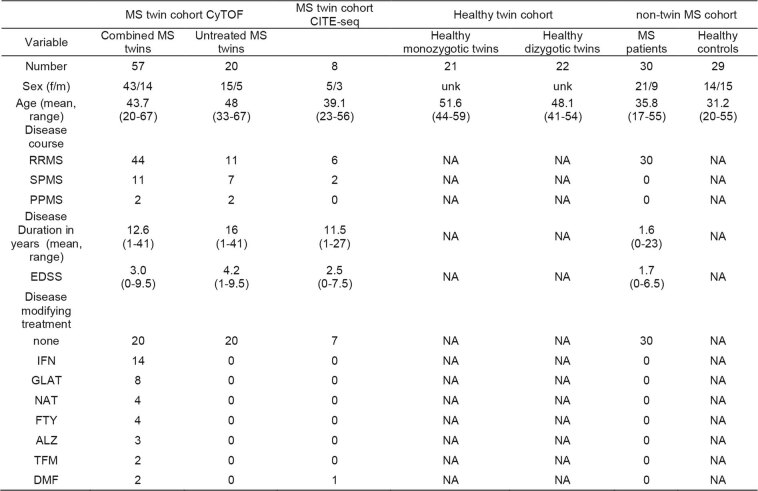
Clinical and demographic baseline characteristics of all study cohorts

Overview of baseline characteristics of the groups and cohorts analyzed in the present study.

MS = Multiple Sclerosis; ALZ = Alemtuzumab; DMF = Dimethyl Fumarate; FTY = Fingolimod; GLAT = Glatiramer Acetate; IFN = beta Interferons; NAT = Natalizumab; TFM = Teriflunomide; f = female; m = male; RRMS = relapsing-remitting MS; SPMS = secondary progressive MS; PPMS = primary progressive MS; NA = not applicable; unk = unknown.

**Extended Data Table 2:**
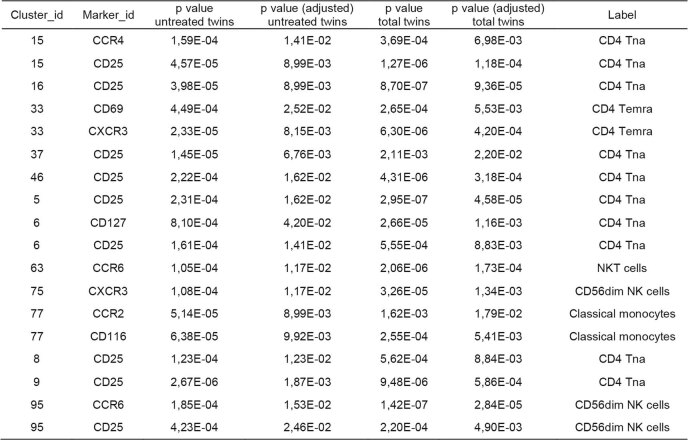
Overview of results for data-driven analysis of mass cytometry data using diffcyt

Shown are cluster-marker combinations that were statistically significant (p adj < 0.05) in two comparisons: 1) comparing all MS-affected twins to their unaffected twin siblings and 2) comparing only untreated MS-affected twins to unaffected siblings using the moderated *limma-trend* method implemented in *diffcyt* with a false-discovery correction according to the Benjamini-Hochberg approach.

Tna = naive T cells; Temra = effector memory T cells re-expressing CD45RA.

## Data Availability

Raw mass cytometry data can be accessed at 10.17632/fzs5ph5p8s.1. CITE-seq data are available at 10.17632/278fy5m2yj.2. Publicly available flow cytometry data of healthy monozygotic and dizygotic twin pairs^17^ were accessed at http://www.tinyurl.com/twinsFACSdata. The publicly available cross-sectional MS mass cytometry dataset for non-twins^14^ was accessed at http://flowrepository.org/experiments/2166/.

## Code availability

The code for the mass cytometry analysis and the variance component analysis of the healthy monozygotic and dizygotic twin pairs is available at https://github.com/florianingelfinger/MStwins. The code for the CITE-seq analysis of the cohort for twins with MS can be accessed at https://github.com/beltranLab/twin_study_Nature_2021.
