# Supplementary material for: Twin study reveals non-heritable immune perturbations in multiple sclerosis
Source: Nature. 2022 Feb 16;603(7899):152–8. doi: 10.1038/s41586-022-04419-4 (PMC8891021; doi:10.1038/s41586-022-04419-4)
Supplement: Supplementary file 1 — Reporting Summary [file 41586_2022_4419_MOESM1_ESM.pdf]

nature research

Corresponding author(s): Prof. Dr. Burkhard Becher

Last updated by author(s): Dec 20, 2021

Reporting Summary

Nature Research wishes to improve the reproducibility of the work that we publish. This form provides structure for consistency and transparency in reporting. For further information on Nature Research policies, see our Editorial Policies and the Editorial Policy Checklist.

Statistics

For all statistical analyses, confirm that the following items are present in the figure legend, table legend, main text, or Methods section.

n/a	Confirmed	
☐	☒	The exact sample size (*n*) for each experimental group/condition, given as a discrete number and unit of measurement
☐	☒	A statement on whether measurements were taken from distinct samples or whether the same sample was measured repeatedly
☐	☒	The statistical test(s) used AND whether they are one- or two-sided<br>*Only common tests should be described solely by name; describe more complex techniques in the Methods section.*
☐	☒	A description of all covariates tested
☐	☒	A description of any assumptions or corrections, such as tests of normality and adjustment for multiple comparisons
☐	☒	A full description of the statistical parameters including central tendency (e.g. means) or other basic estimates (e.g. regression coefficient) AND variation (e.g. standard deviation) or associated estimates of uncertainty (e.g. confidence intervals)
☐	☒	For null hypothesis testing, the test statistic (e.g. *F*, *t*, *r*) with confidence intervals, effect sizes, degrees of freedom and *P* value noted<br>*Give P values as exact values whenever suitable.*
☒	☐	For Bayesian analysis, information on the choice of priors and Markov chain Monte Carlo settings
☐	☒	For hierarchical and complex designs, identification of the appropriate level for tests and full reporting of outcomes
☐	☒	Estimates of effect sizes (e.g. Cohen's *d*, Pearson's *r*), indicating how they were calculated

Our web collection on statistics for biologists contains articles on many of the points above.

Software and code

Policy information about availability of computer code

Data collection	Fluidigm CyTOF was used to facilitate mass cytometry acquisition.
Data analysis	R version 3.6.1 and 4.0.1 was used in RStudio 1.3.959 and Visual Studio Code 1.63 to analyze all data presented. FlowJo 10.6 was used to preprocess the data. Cell Ranger 6.1 was used to process raw sequencing reads. For downstream analysis the following packages were used as indicated inn the method section of the manuscript: umap (v0.2.6), FlowSOM (v1.17.4), ConsensusClusterPlus (v1.52.0), diffcyt (v1.10.0), grappolo (v0.5.1), vite (v0.4.10), igraph (v1.2.7), ggraph (v2.0.5), pheatmap (v1.0.12), Hmisc (v4.4.0), corrplot (v0.84), ggplot2 (v3.3.5), Seurat (v4.0.3), DoRothEA (v1.2.2), Monocle 3 (v0.2.3.0), SCANPY (1.6.0), mgcv (v1.8-31), umx (v4.10.10), stats (v4.0.1), rstatix (v0.7.0).

For manuscripts utilizing custom algorithms or software that are central to the research but not yet described in published literature, software must be made available to editors and reviewers. We strongly encourage code deposition in a community repository (e.g. GitHub). See the Nature Research guidelines for submitting code & software for further information.

Data

Policy information about availability of data

All manuscripts must include a data availability statement. This statement should provide the following information, where applicable:

- Accession codes, unique identifiers, or web links for publicly available datasets
- A list of figures that have associated raw data
- A description of any restrictions on data availability

Raw mass cytometry data can be accessed at doi:10.17632/fzs5ph5p8s.1. CITE-seq data is available at doi:10.17632/278fy5m2yj.1. Code used for the CITE-seq analysis is accessible at https://github.com/beltranLab/twin_study_Nature_2021. Code used for the mass cytometry and variance component analysis is presented at https://github.com/florianingelfinger/MStwins.

Field-specific reporting

Please select the one below that is the best fit for your research. If you are not sure, read the appropriate sections before making your selection.

☒ Life sciences ☐ Behavioural & social sciences ☐ Ecological, evolutionary & environmental sciences

For a reference copy of the document with all sections, see nature.com/documents/nr-reporting-summary-flat.pdf

Life sciences study design

All studies must disclose on these points even when the disclosure is negative.

Sample size	The MS TWIN STUDY has been established in 2012 and has currently enrolled 87 monozygotic twin pairs with discordance for MS. Samples for the presented MS twin cohort analysis have been selected from the MS TWIN STUDY with respect to availability of sufficient biomaterial (PBMCs samples) until May 2018 (CyTOF) and May 2020 (additional samples for CITE-seq).
Data exclusions	Samples containing less than 100 cells and the data of the corresponding co-twin were excluded in mass cytometry analysis. For CITE-seq only samples with data for both the MS-affected and unaffected sibling were used for downstream analysis. These criteria have been pre-established to ensure a reliable quantification of immune cell frequencies and median marker expressions.
Replication	Validation of the finding in the MS twin cohort have been carried out using CITE-seq of additional twin pairs in the MS twin cohort and using publicly available mass cytometry data of a cross-sectional non-twin MS cohort. Mass cytometry was acquired in two independent runs and was performed once due to limited sample availability of this precious study cohort. CITE-seq analysis was performed once.
Randomization	Twin samples were not allocated to experimental groups as this was an observational study with pre-established clinical conditions (MS, nonn-MS). However, randomization was carried out to balance the two individual runs of the mass cytometry analysis. For twin pairs were equally randomized and the respective co-twin was analyzed in the same batch as the MS-affected twin.
Blinding	Investigators were blinded during the experiments. After data acquisition and preprocessing unblinding was necessary to perform group stratification. However, the unbiased nature of the analysis approach prevented investigator bias.

Reporting for specific materials, systems and methods

We require information from authors about some types of materials, experimental systems and methods used in many studies. Here, indicate whether each material, system or method listed is relevant to your study. If you are not sure if a list item applies to your research, read the appropriate section before selecting a response.

Materials & experimental systems

n/a	Involved in the study	
☐	☒	Antibodies
☒	☐	Eukaryotic cell lines
☒	☐	Palaeontology and archaeology
☒	☐	Animals and other organisms
☐	☒	Human research participants
☒	☐	Clinical data
☒	☐	Dual use research of concern

Methods

n/a	Involved in the study	
☒	☐	ChIP-seq
☒	☐	Flow cytometry
☒	☐	MRI-based neuroimaging

Antibodies

Antibodies used	All mass cytometry antibodies used in the study including manufacturer, clone, heavy metal tag and dilutions are listed in Supplementary Table 3. CITE-seq antibodies are listed in Supplementary Table 7.
Validation	Heavy metal conjugated antibodies have been validated by assessing the respective expression pattern in a fully stained sample across cell types in the human peripheral blood and comparing it to the manufacturers technical sheets and cytometry and sequencing data available in the literature. Clones have been preferentially chosen based on previous experience with the respective antibody for flow cytometry.

Human research participants

Policy information about studies involving human research participants

Population characteristics	Inclusion criteria for study participation were met if in one co-twin of monozygotic twins an MS diagnosis according to the revised McDonald criteria was established, whereas the co-twin was clinically healthy. Exclusion criteria were infection as well as treatment with antibiotics or high dose intravenous glucocorticosteroids within three months prior to sampling. Monozygotic twin pairs clinically discordant for MS (n=61) visited the outpatient department at the Institute of Clinical

Neuroimmunology at the LMU Klinikum Munich for a detailed interview, neurological examination, blood sampling and MRI investigations (in a proportion of twins only). To confirm MS diagnosis, medical records including MRI scans were obtained and reviewed (Extended Data Table 1, Supplementary Tables 1-2). 43 female and 14 male twin pairs were analyzed by mass cytometry and age ranged between 20 and 67 years (mean = 43.7 years). For CITE-seq analysis 5 female and 3 male twin pairs at an age between 23 and 56 years (mean = 39.1 years) were analyzed.

Recruitment

The MS twin cohort is part of the MS TWIN STUDY and presents a cohort of monozygotic twins with discordance for MS and is located at the Institute of Clinical Neuroimmunology at the LMU Klinikum Munich, Germany. Recruitment started in May 2012 and is still ongoing, samples used in the present study were collected up to May 2018. Information regarding the MS TWIN STUDY is distributed via an internet appeal to people with MS (such as webpages or magazines of national and regional MS societies, support groups) or to MS neurologists via publications and talks from the PIs from the MS TWIN STUDY. Contact with interested participants is based on a voluntary approach by the twins or a referring neurologist. Recruitment is planned after detailed information is provided during a detailed telephone interview. Potential biases of a study based on a voluntary appeal in contrast to a registry based approach are as follows: Due to limitations to take the trouble to travel to the study site our study cohort might have a larger fraction of younger twins with less severe disease severity whereas people with higher disease severity or older age refuse to take the trouble to travel to the study centre. In the same line a potential bias reflects the outreach of an internet appeal to younger people. In addition, the appeal for a voluntary participation in a twin study to unravel the triggers for MS might attract participants with more than one family member affected with MS and might introduce a higher familial risk in the study cohort than in the general MS population. Another limitation is the heterogeneity of the cohort regarding age, disease course and varying treatments. Disease modifying treatment of the MS affected twin within a twin pair presents the most important bias and this limitation cannot be overcome since most people with MS receive disease modifying treatment and the repertoire of effective treatments has been ever expanding. But as explained in detail these pairs have been excluded for the relevant analyses. However, taking these potential selection biases into account we do not assume that these impact our study results on the immune perturbation in MS.

Ethics oversight

The MS TWIN STUDY was approved by the local ethics committees of the Ludwig-Maximilians-University of Munich (ethics approval project number 267-13). All participants gave written informed consent, according to the principles of the Declaration of Helsinki.

Note that full information on the approval of the study protocol must also be provided in the manuscript.
